# High-Precision Pest Management Based on Multimodal Fusion and Attention-Guided Lightweight Networks

**DOI:** 10.3390/insects16080850

**Published:** 2025-08-16

**Authors:** Ziye Liu, Siqi Li, Yingqiu Yang, Xinlu Jiang, Mingtian Wang, Dongjiao Chen, Tianming Jiang, Min Dong

**Affiliations:** 1 China Agricultural University, Beijing 100083, China; 2 National School of Development, Peking University, Beijing 100871, China; 3 Department of Electronic and Communication Engineering, Beijing Electronic Science and Technology Institute, Beijing 100070, China; 4 College of Plant Protection, China Agricultural University, Beijing 100083, China

**Keywords:** robust pest management models, pest and natural enemy monitoring, agricultural visual intelligence, edge computing in agriculture, environmental sensor-guided detection

## Abstract

In the context of global food security and sustainable agricultural development, the efficient recognition and precise management of agricultural insect pests and their predators have become critical challenges in the domain of smart agriculture. To address the limitations of traditional models that overly rely on single-modal inputs and suffer from poor recognition stability under complex field conditions, a multimodal recognition framework has been proposed. This framework integrates RGB imagery, thermal infrared imaging, and environmental sensor data. A cross-modal attention mechanism, environment-guided modality weighting strategy, and decoupled recognition heads are incorporated to enhance the model’s robustness against small targets, intermodal variations, and environmental disturbances. Evaluated on a high-complexity multimodal field dataset, the proposed model significantly outperforms mainstream methods across four key metrics, precision, recall, F1-score, and mAP@50, achieving 91.5% precision, 89.2% recall, 90.3% F1-score, and 88.0% mAP@50. These results represent an improvement of over 6% compared to representative models such as YOLOv8 and DETR. Additional ablation studies confirm the critical contributions of key modules, particularly under challenging scenarios such as low light, strong reflections, and sensor data noise. Moreover, deployment tests conducted on the Jetson Xavier edge device demonstrate the feasibility of real-world application, with the model achieving a 25.7 FPS inference speed and a compact size of 48.3 MB, thus balancing accuracy and lightweight design. This study provides an efficient, intelligent, and scalable AI solution for pest surveillance and biological control, contributing to precision pest management in agricultural ecosystems.

## Simple Summary

This study focuses on applying cross-modal fusion to multi-source agricultural perception tasks and proposes a unified detection framework integrating cross-modal attention-guided feature fusion, an environment-guided modality attention mechanism, and a decoupled dual-target detection head. The framework enables efficient collaborative modeling of visible light, infrared, and environmental sensor data. In multi-class pest and predator recognition tasks, the proposed method consistently achieves diagonal values in the range of 0.73–0.79 in the confusion matrix across five categories, while significantly outperforming mainstream models such as YOLOv5, YOLOv8, RetinaNet, and Faster R-CNN in overall precision, recall, and generalization capability. Experimental results demonstrate that the framework offers strong feature discrimination and cross-modal information integration in complex field scenarios, providing robust technical support for precision ecological monitoring in smart agriculture.

## 1. Introduction

Driven by the rapid advancement of digital agriculture and intelligent perception technologies, smart agriculture has increasingly become a pivotal means of improving agricultural productivity and ensuring food security [1,2]. Among these technologies, field pest monitoring and precision control based on visual and sensor data have emerged as core components, demonstrating promising potential within ecological and sustainable agricultural systems [2]. Among various green pest control approaches, biological control using predators has been widely adopted due to its high efficiency, ecological friendliness, and minimal environmental pollution [3,4]. However, achieving accurate identification of pests and their predators in complex outdoor environments remains a significant challenge, particularly under conditions involving high visual similarity, small object scales, and limited annotated samples. Traditional recognition models often fail to meet practical demands in such scenarios. Most existing approaches rely heavily on RGB images for detection or classification tasks. Although substantial progress has been made using deep learning models such as YOLO, Faster R-CNN, and ResNet [5,6,7,8], their performance in real-world field environments is frequently hindered by varying lighting conditions, background occlusion, imaging angles, and motion blur [9]. In particular, high misclassification rates persist when distinguishing predators with appearances similar to pests, compromising the scientific formulation of control strategies and the ecological balance. Furthermore, the characteristics of pest–predator targets—such as small body size, low occurrence frequency, and annotation difficulty—further limit model performance under supervised learning paradigms [10]. Consequently, enhancing robustness to environmental perturbations and improving discriminative capacity through multimodal data integration have become critical research directions in smart agriculture target recognition.

In recent years, multimodal learning has achieved notable success in computer vision and remote sensing by jointly modeling heterogeneous sensor data, thereby improving recognition accuracy under non-ideal conditions [11]. Within the agricultural domain, several studies have explored the use of infrared images, thermal imaging, radar signals, and environmental sensor data for tasks such as pest detection and crop monitoring [12]. However, most of these efforts have focused on static crop characterization, with few targeting individual animal recognition in field conditions. Moreover, there is a lack of standardized datasets and deployment-oriented model architectures. Semantic disparities, inconsistent signal-to-noise ratios, and misaligned attention distributions among modalities frequently lead to information conflicts or redundancy, highlighting the need for fusion mechanisms capable of semantic alignment and adaptive modality selection to fully exploit the synergy of multimodal data.

The primary purpose of this study is to develop and validate a high-precision, environmentally robust multimodal sensor fusion framework for field pest recognition under real-world agricultural conditions. To this end, RGB images, thermal infrared imaging, and environmental sensor data including temperature, humidity, and light intensity are integrated. A multi-branch deep fusion network featuring cross-modality alignment is designed. This architecture incorporates modality attention and reweighting strategies to dynamically adjust the importance of each modality, mitigating biases under weak supervision and improving overall discriminative power and environmental adaptability. The main contributions of this study are summarized as follows:

1. A multi-branch feature fusion network with cross-modal alignment is developed, wherein RGB and IR image features are extracted through dedicated visual encoders. A cross-modal attention mechanism is introduced to align semantic regions across modalities, enhancing fusion quality and reducing semantic inconsistency.
2. An environment-guided modality selection and reweighting mechanism is constructed using real-time temperature, humidity, and illumination data. A modality scheduling sub-network dynamically adjusts the fusion weights of RGB and IR channels based on environmental conditions, improving model robustness and adaptability in extreme weather or complex lighting scenarios.
3. A decoupled recognition head is introduced to support joint detection of pest and predator targets. This structure addresses issues related to category imbalance and semantic confusion by employing separate detection and classification branches, thereby reducing cross-category misclassification and improving overall joint recognition accuracy.
4. The code and datasets supporting this study are publicly available on GitHub to facilitate reproducibility and collaboration in the field (access date: 15 August 2025): https://github.com/Aurelius-04/pestdetection.

The remainder of this paper is organized as follows. Section 2 surveys related work, covering field pest and natural enemy recognition, multimodal sensor fusion in agriculture, and recent advances in deep fusion and attention mechanisms. Section 3 details the materials and methods, including dataset construction, preprocessing and augmentation, and the proposed multimodal framework with cross-modal attention, environment-guided modality weighting, and a decoupled detection head. Section 4 presents the results and discussion, encompassing the experimental setup, comparisons with strong baselines, comprehensive ablation studies, modality ablation, edge deployment efficiency on Jetson Xavier, and limitations with practical implications. Section 5 concludes this study by summarizing key findings and outlining directions for future research.

## 2. Related Work

### 2.1. Pest Target Detection Methods

In recent years, with the rapid advancement of deep learning, CNN-based object detection methods have been widely applied to agricultural pest identification. Faster R-CNN, with its two-stage detection paradigm, remains a representative model for fine-grained pest recognition. For example, Ali et al. (2023) developed the Faster-PestNet framework with an optimized region proposal network and multi-scale feature extraction, achieving strong results on IP102 and local datasets [13]. To improve detection efficiency, YOLO series models have attracted extensive attention for their high inference speed and end-to-end architecture. Early lightweight variants, such as Tang et al. (2023) based on YOLOv5, improved detection speed and reduced computation while maintaining competitive accuracy [14]. More recent work has achieved notable performance gains: Yin et al. (2024) proposed an improved YOLOv8 with M-CBAM attention, MPDIoU loss, and Ghost convolution, reaching 95.8% AP with only 2.15 M parameters [15]; Dong et al. (2024) developed PestLite, integrating MTSPPF pooling, advanced attention mechanisms, and CARAFE upsampling, improving mAP50 from 87.9% to 90.7% while reducing parameters [16]; and Huang et al. (2025) introduced YOLO-YSTs, an enhanced YOLOv10n for yellow sticky trap images, achieving an mAP50 of 86.8% and 139 FPS on edge devices [17]. More recently, Transformer-based approaches have been explored to leverage global context modeling. Zhang and Lv (2024) proposed TinySegformer, a lightweight segmentation model with sparse attention and quantization, achieving high accuracy and 32.7 FPS on edge devices [9]. Liu et al. (2025) further enhanced detection accuracy through Feature Representation Compensation (FRC) and Regional Pale-shaped Self-Attention (RPSA), surpassing state-of-the-art CNN methods by 4.5% and 5.7% mAP on IP102 and FPD datasets, respectively [18].

Compared to pest detection, research on agricultural predators has emerged more recently, with limited publicly available datasets. Existing studies primarily focus on image classification and a few detection tasks. Due to their high visual similarity to pests, smaller body size, and lower frequency of appearance, predators are difficult to distinguish using traditional appearance-based recognition methods. In studies on insect co-occurrence image recognition, Wang et al. (2022) noted that substantial visual overlap between pests and predators often leads to misclassification by current models. The lack of labeled data and category annotations further restricts the discriminative capabilities of deep models [19]. Therefore, a new recognition framework that addresses data acquisition, model architecture, and robustness enhancement is urgently needed to meet the challenges of joint pest–predator identification in real-world field environments.

### 2.2. Applications of Multimodal Sensor Fusion in Agricultural Contexts

Multimodal sensor technologies have been widely adopted in modern agriculture to enable intelligent decision-making tasks such as crop condition monitoring, disease detection, and yield estimation by integrating visual imagery with environmental data [20]. RGB imagery, as the most common visible light modality, contains rich texture and color information and is broadly used for visual characterization of crops and detection of pests and diseases. Simultaneously, thermal infrared imaging, due to its sensitivity to temperature changes, is employed in scenarios such as crop water stress monitoring, lesion detection, and greenhouse microclimate regulation. For instance, Zhao et al. (2025) developed an early tomato disease diagnostic system based on the fusion of visible and infrared imaging, which significantly improved lesion discrimination performance [21]. On the environmental data layer, parameters such as temperature, humidity, and light intensity can be continuously recorded using low-cost sensors, providing crucial contextual information for modeling growth conditions and crop stress. Qiu et al. (2025) introduced an intelligent fertilization robot system integrating image acquisition, environmental monitoring, and wireless communication, enabling multidimensional physiological data collection from field crops and alleviating issues such as resource waste, environmental pollution, and low yield associated with traditional fertilization practices [22]. Although these studies highlight the potential of multimodal sensors at the crop level, existing research primarily focuses on plant growth status, foliar disease detection, or macroecological factor analysis. Very few efforts have addressed fusion-based recognition of agricultural animal individuals. Most current approaches limit perception to static objects and rely heavily on texture or color features, lacking comprehensive modeling of the behavior and status of dynamic targets such as pests and predators. In natural field environments, individual animals frequently present challenges such as strong occlusion, significant scale variation, and high background confusion, making single-modality information insufficient for effective recognition and classification. Furthermore, a lack of multimodal datasets and unified modeling frameworks for pest–predator co-occurrence ecological scenarios has restricted the deeper application of multimodal fusion technologies in ecological regulation.

To address these limitations, a multimodal sensor fusion recognition framework has been proposed for joint pest and predator identification in field environments. For the first time, RGB imagery, thermal infrared imaging, and environmental sensor data, including temperature, humidity, and light intensity, have been integrated into a deep fusion network to enable cooperative modeling of distinct biological targets. Compared with traditional plant-centric fusion strategies, this study emphasizes fine-grained perception and class-level discrimination of agricultural animals, offering a novel perspective and technical pathway for extending multimodal recognition methods within complex ecological systems.

### 2.3. Advances in Deep Fusion and Attention Mechanisms for Multimodal Learning

Multimodal fusion serves as a key technology for jointly modeling heterogeneous data sources and has been widely applied in image understanding, semantic segmentation, video analysis, and sensor fusion tasks [23]. Based on the timing and method of fusion, current strategies can be categorized into early fusion, mid-level fusion, and late fusion. Early fusion concatenates or aligns input-level data and is suitable for tasks with structurally similar and semantically close modalities, such as RGB and near-infrared (NIR) image fusion. Mid-level fusion introduces cross-modal interaction layers during feature extraction to align semantic spaces and enhance representations across modalities, often through shared attention mechanisms in dual-branch networks. Late fusion focuses on combining independent modality-specific decisions at the output level, which is particularly beneficial when modalities differ significantly in characteristics or when limited training data is available. Among these, mid-level fusion has recently gained traction due to its balance between expressive capacity and interactive flexibility. The introduction of Transformer architectures has substantially advanced cross-modal attention mechanisms, providing powerful tools for deep semantic modeling. ViLT removes the visual encoder and directly embeds image patches into a Transformer architecture to interact with textual features, significantly improving pretraining efficiency [24]. The METER framework integrates symmetric cross-modal Transformer modules within a dual-stream vision–language structure, enhancing adaptability and transferability across downstream tasks [25]. Sharma et al. employed a Co-Attention Transformer to explicitly model bidirectional attention pathways between image and text modalities, enabling fine-grained region-level interaction [26]. These methods typically leverage modality attention, dynamic weighting, and channel selection mechanisms to demonstrate robust performance under conditions with noisy modalities, large semantic gaps, or incomplete inputs. Modality reweighting, in particular, is highly applicable to scenarios involving image and sensor data fusion, as it allows the importance of each modality to be adaptively adjusted based on task context and input quality, thereby mitigating redundancy and bias.

Despite these achievements in general vision tasks, applications in agricultural contexts—especially those involving fusion of multi-source sensors and unstructured data—remain in early exploratory stages. Most current agricultural multimodal studies rely on fixed fusion structures or static weighting schemes, which are inadequate for coping with the dynamic changes and heterogeneous noise present in field environments [27,28]. Studies that jointly model crop and environmental information often overlook structural asymmetry and semantic gaps among modalities, limiting the effectiveness of fusion. The proposed multimodal recognition framework draws upon principles of cross-modal attention and dynamic reweighting. By incorporating RGB imagery, thermal infrared imaging, and environmental sensor data, the framework introduces learnable modality attention and feature reweighting modules, offering an adaptive and interpretable fusion strategy tailored for pest–predator recognition. This contributes to extending the application frontier of multimodal learning within intelligent agricultural perception systems.

## 3. Materials and Method

### 3.1. Data Collection

**Figure 1:**
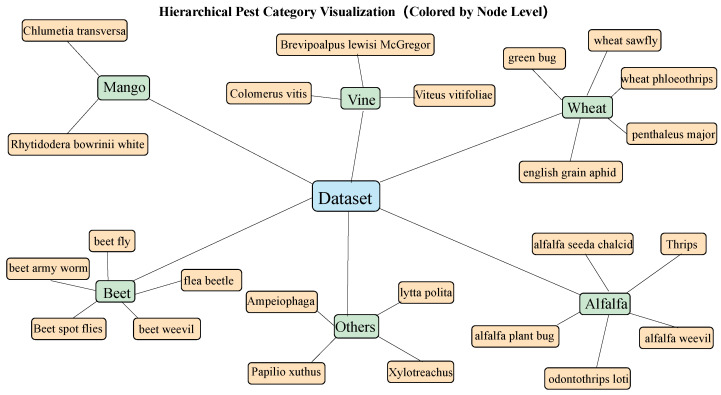
Hierarchical visualization of pest categories in the dataset. The graph adopts a three-level structure where the central blue node represents the entire dataset, green nodes indicate primary host crop categories (e.g., rice, wheat, and citrus), and orange nodes denote individual pest species associated with each crop. This color-coded hierarchy enhances interpretability and reflects the pest–host relationships in agricultural contexts.

In this study, a multimodal field dataset for pest and natural enemy co-recognition was constructed by deploying multisensor acquisition platforms in representative agricultural test fields located in Bayannur, Inner Mongolia Autonomous Region, and Weifang, Shandong Province, and using Internet data [29], from May to October 2023. These deployment sites were selected to encompass varying latitudes, climatic conditions, and cropping systems, as shown in Table 1 and Table 2 and Figure 1. For visual data acquisition, high-definition RGB industrial cameras with a resolution of 1920 × 1080 and FLIR One Pro infrared thermal imagers with a resolution of 640 × 480 were synchronously installed. A hardware-triggering mechanism was employed to ensure simultaneous capture of RGB and infrared images at a frame rate of 5 frames per minute, thereby preserving consistency in image content and completeness in thermal characteristics. The dataset covers various time periods (morning, noon, and evening) and weather conditions (sunny, cloudy, and foggy), enabling the capture of natural lighting variation and infrared response changes that influence target recognition. To enhance the model’s ability to adapt to dynamic environmental conditions, the acquisition system integrated DHT22 sensors for temperature and humidity and LDR sensors for light intensity. For reliable long-term operation in the field, the DHT22 sensors were placed inside IP65-rated ventilated protective housings to shield them from rain, dust, and direct sunlight while allowing adequate airflow for accurate readings. The sensors were mounted on the underside of the acquisition platform at a height of 1.5 m above the ground to minimize direct water exposure and soil splash during precipitation events. These sensors recorded ambient data at a frequency of one reading per second, which were temporally aligned with the corresponding image frames. This relatively high sampling frequency was chosen to capture rapid microclimatic fluctuations—such as sudden temperature drops, humidity spikes, or light intensity changes—that may occur due to wind gusts, passing clouds, or irrigation events. Such fine-grained temporal resolution ensures accurate matching of sensor readings with each image frame for precise multimodal fusion. Furthermore, the recorded data packets are small (tens of bytes per reading), and transmission is handled over a local Wi-Fi network to the edge unit, making the additional network load negligible. In addition to the six main pest classes, the dataset also includes two common predator classes, namely lady beetles and lacewings. These predators share certain visual and habitat similarities with some pests in field environments, and their inclusion in the annotations helps the model learn clearer category boundaries during feature representation.

Only image–sensor pairs that met strict quality and relevance criteria were retained for model training and evaluation. Specifically, RGB and thermal images were required to be captured within the same hardware trigger cycle to guarantee precise temporal alignment, and their corresponding environmental sensor readings had to be complete and valid without missing or corrupted values. In addition, at least one target object (pest or predator) was required to be clearly visible and occupy a minimum of 20 × 20 pixels in the RGB image to avoid ambiguity during labeling. Samples with excessive motion blur, severe occlusion, or any sensor malfunction were excluded. Consequently, although the acquisition system operated at a nominal rate of 5 frames per minute for images and 1 Hz for environmental data, the counts in Table 1 reflect only the valid samples remaining after rigorous cleaning and filtering. This explains the difference between RGB and infrared image counts, as well as the smaller-than-expected total relative to the theoretical capture frequency. This selection process ensured that the dataset was both high-quality and representative of real-world conditions, thereby improving the reliability and objectivity of the experimental results.

### 3.2. Data Preprocessing and Augmentation

In multimodal recognition tasks, raw data often suffer from occlusion, illumination perturbation, asynchronous sampling, and inconsistent modality scales, which severely compromise the model’s stability and generalization in complex field environments. To enhance the robustness of the multimodal framework, systematic preprocessing and augmentation strategies were designed separately for image data and environmental sensor data. To improve tolerance to partial occlusion, an occlusion simulation [30] operation was introduced by randomly masking rectangular regions in the input images to mimic vegetation blockage or target defocus, formally defined as follows:

$$I^{\prime }\left(x,y\right)=\left\{\begin{array}{cc} 0, & (x,y)\in R\\ I(x,y), & \mathrm{otherwise}, \end{array}\right.$$

where I(x,y) denotes the original image intensity at pixel location (x,y) and R represents the randomly selected occluded region whose pixel values are set to zero to simulate missing information. To enhance adaptability under varying illumination, Gaussian perturbations were applied to the brightness channel of the image [31], simulating natural lighting fluctuations, expressed as follows:

$$I^{\prime \prime }=I+N\left(0,\sigma^{2}\right),$$

where $I^{\prime \prime }$ represents the perturbed image and $N(0,\sigma^{2})$ denotes zero-mean Gaussian noise with variance $\sigma^{2}$, modeling image uncertainty under brightness variation. To address the temporal misalignment between sensor data and image frame rates, linear interpolation was employed to resample temperature [32], humidity, and light intensity time series, ensuring alignment with image timestamps. The interpolation formula is defined as follows:

$${\hat{s}}_{j}=s_{k}+\frac{s_{k+1}-s_{k}}{t_{k+1}-t_{k}}\cdot \left(t_{j}^{\prime }-t_{k}\right),\;t_{k}\leq t_{j}^{\prime }<t_{k+1},$$

where ${\hat{s}}_{j}$ is the interpolated sensor value, $s_{k}$ and $s_{k+1}$ are the original observations at adjacent sampling points, $t_{j}^{\prime }$ is the target image frame timestamp, and $t_{k}$, $t_{k+1}$ are the surrounding sensor timestamps. After interpolation, normalization [33] was applied to all sensor data to unify the value scales of multimodal inputs, expressed as follows:

$$s_{j}^{\mathrm{norm}}=\frac{s_{j}-s_{\min}}{s_{\max}-s_{\min}},$$

where $s_{j}^{\mathrm{norm}}$ denotes the normalized value and $s_{\min}$, $s_{\max}$ represent the minimum and maximum values of the corresponding modality in the training set. Finally, a unified encoding structure was constructed to integrate RGB, thermal, and environmental modalities by independently encoding each input and concatenating their feature representations, thereby achieving cross-modal semantic fusion. Specifically, the RGB image $I_{j}$ is processed by the RGB feature encoder $f_{\mathrm{rgb}}\left(\cdot \right)$, the thermal infrared image $T_{j}$ is processed by the thermal feature encoder $f_{\mathrm{thermal}}\left(\cdot \right)$, and the environmental sensor vector $s_{j}^{\mathrm{norm}}$ is processed by the environmental encoder $f_{\mathrm{env}}\left(\cdot \right)$. The resulting modality-specific features are concatenated to form the unified multimodal representation:

$$X_{j}=[\;f_{\mathrm{rgb}}\left(I_{j}\right)\;∥\;f_{\mathrm{thermal}}\left(T_{j}\right)\;∥\;f_{\mathrm{env}}\left(s_{j}^{\mathrm{norm}}\right)\;],$$

where $X_{j}$ is the fused representation of the *j*-th sample, $f_{\mathrm{rgb}}\left(\cdot \right)$ denotes the RGB feature encoder, $f_{\mathrm{thermal}}\left(\cdot \right)$ denotes the thermal feature encoder, $f_{\mathrm{env}}\left(\cdot \right)$ denotes the environmental feature encoder, and ∥ indicates feature concatenation. This unified encoding strategy preserves modality-specific characteristics while enabling joint optimization in subsequent fusion and recognition stages.

### 3.3. Proposed Method

#### 3.3.1. Overall

**Figure 2:**
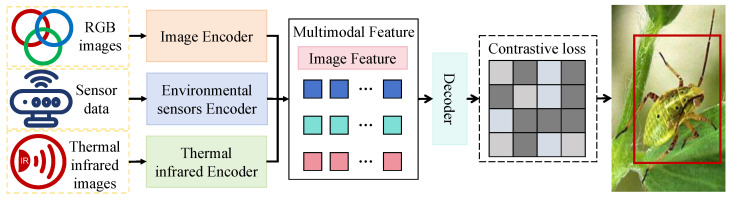
Overview of the proposed multimodal recognition framework. The architecture consists of three input branches: RGB images are processed by the image encoder to extract visual semantic features; thermal infrared images are encoded by the thermal encoder to capture thermal intensity patterns; and environmental sensor data are encoded to provide contextual environmental cues.

The proposed multimodal pest recognition framework, as illustrated in Figure 2, adopts a multi-input, multi-branch end-to-end neural architecture designed to integrate RGB images, infrared thermal maps, and environmental sensor data for precise detection and localization of pests and predators in field environments. The preprocessed multimodal data are first fed into three independent encoder modules: spatial–semantic joint features are extracted from RGB images via a visual backbone network (image encoder); thermal intensity and structural cues are derived from infrared images using a dedicated thermal encoder; and environmental signals such as temperature, humidity, and illumination are encoded into high-dimensional modality-guided vectors through an environmental sensor encoder. These three types of features are subsequently combined within a multimodal fusion module. A cross-modal attention mechanism is employed to spatially align regions between RGB and thermal modalities, enhancing the expression of semantically complementary areas. Simultaneously, environment-guided attention adaptively adjusts the fusion weights between RGB and thermal features based on external conditions, enabling the model to dynamically balance modality contributions under diverse environmental settings. The fused features are then passed to a Transformer-based decoder for contextual modeling and query generation, which outputs a set of multi-label embeddings representing potential targets. Finally, a contrastive loss is applied to reinforce semantic discrimination between pest and predator categories, and a detection head is used to predict bounding boxes and class labels for each identified instance in the image. This architecture demonstrates the effectiveness of multimodal collaborative modeling and cross-modal feature alignment, leading to significantly improved recognition performance in complex and dynamic field scenarios. To reduce confusion between pests and visually similar predators, our detection framework includes 6 pest classes and 2 predator classes and incorporates a contrastive loss. This loss uses “pest–predator” category labels as supervision to maximize inter-class distances and minimize intra-class distances in the feature embedding space, thereby improving pest recognition accuracy and reducing false positives. The predator classes are lady beetles and lacewings.

#### 3.3.2. Cross-Modal Attention-Guided Feature Fusion Module

**Figure 3:**
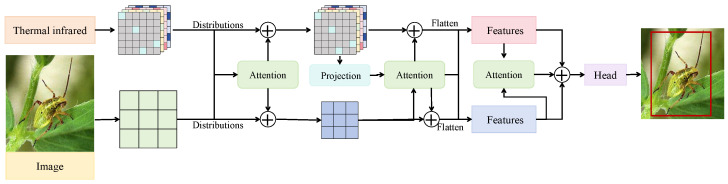
Schematic diagram of the cross-modal attention-guided feature fusion module. The module integrates RGB and thermal infrared modalities through projection-based attention alignment and feature interaction, followed by flattening, densification, and fusion operations to generate robust multimodal detection outputs.

The cross-modal attention-guided feature fusion module, as shown in Figure 3, is designed to address the discrepancies in semantic distribution and information representation between RGB images and infrared thermal maps, thereby enhancing the discriminative capability of the fused features for pest and predator targets. This module comprises four key components: a modality aligner, a multi-scale projection fusion block, a cross-modal attention unit, and a final feature integration path. RGB and infrared images are independently passed through two shallow convolutional encoders to extract intermediate features of size C×H×W, where C=64 and H=W=64. Based on these features, distribution estimation is conducted using histogram-based kernel density approximation to capture spatial energy distributions across modalities. The Kullback–Leibler divergence [34], defined as $D_{KL}\left(P_{rgb}|P_{ir}\right)=\sum_{i}P_{rgb}\left(i\right)\log\frac{P_{rgb}\left(i\right)}{P_{ir}\left(i\right)}$, is used to quantify intermodal inconsistencies at local regions. A set of alignment attention maps is then generated and applied through channel-wise weighting to guide the reconstruction of semantically complementary regions. This process significantly improves the response consistency of both modalities within the same object region and mitigates the edge blur issues frequently observed in infrared imagery. The aligned features are subsequently passed through a multi-scale projection structure, where a 1×1 convolution is applied to reduce the channel dimension to $C^{\prime }=32$. A spatial attention block is then used to identify key intramodal regions, and local cross-modal alignment is performed using the standard attention operation [35] $\mathrm{Softmax}(QK^{T}/\sqrt{d})V$, where *Q* and *K* are derived from the RGB and infrared modalities, respectively, and *V* represents the attended modality. During this process, each feature map is first flattened into a shape of N×C, where N=H×W denotes the number of spatial tokens obtained by unfolding the feature map of height *H* and width *W*, and *C* is the feature channel dimension. This flattened representation is then fed into the bidirectional attention module, resulting in mutually enhanced intermediate feature representations.

To prevent any single modality from dominating the fusion process, a residual gating mechanism is introduced. This mechanism combines the original and attention-enhanced modality features through residual connections and learnable gates, ultimately producing a unified feature map of shape $C^{\prime }\times H\times W$. The fused representation is then integrated with original semantic features in the decoder stage for subsequent object detection and classification. Mathematically, this module explicitly models intermodal distribution discrepancies and incorporates information-theoretic measures to construct a soft alignment mechanism in the visual space. The trainable attention structure further enhances critical region representation. Compared to naive concatenation or averaging, this design adaptively emphasizes thermally salient regions in infrared images, particularly under low illumination, cluttered backgrounds, or occlusion conditions, thereby significantly improving the model’s sensitivity to small predator targets and reducing false detections. Through the coordinated design of cross-modal attention and modality alignment, this module achieves both semantic enhancement and structural unification, providing robust feature support for the overall recognition task.

#### 3.3.3. Environment-Guided Modality Attention Mechanism

To adapt to the influence of dynamic field environments on modality-specific stability and expressive capacity, an environment-guided modality attention mechanism (EMA) is proposed to dynamically modulate the contribution of RGB and infrared image features during multimodal fusion. This mechanism incorporates environmental variables such as temperature, humidity, and illumination as external guidance signals, modeled via a state space network (SSM), which is subsequently coupled with visual features in the spatial domain. Unlike conventional self-attention mechanisms, which primarily capture relative dependencies within input features, EMA leverages exogenous environmental data to establish an explicit modulation gate, enabling guided modality selection and reweighting in the fusion process.

**Figure 4:**
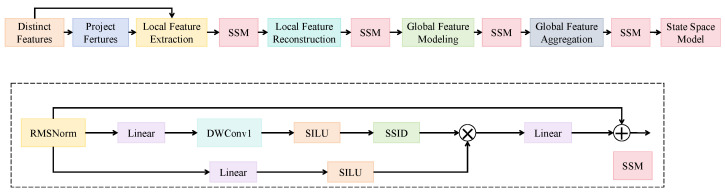
Illustration of the environment-guided modality attention mechanism. This module integrates environmental sensor features such as temperature, humidity, and light intensity to generate attention weights that guide the adaptive fusion of RGB and thermal infrared features.

As shown in Figure 4, the module includes an environment encoder, a local–global state space modeling unit, and a modality fusion block. The environmental vector $x_{e}\in R^{3}$, composed of temperature, humidity, and light intensity, is first projected into a 64-dimensional representation through a linear layer. This is followed by a state space network with a depth of L=3, where each layer consists of RMS normalization, a linear transformation (width 64), depthwise convolution (kernel size 3, stride 1), and a SiLU activation. The core structure, a one-dimensional state space modeling block (SS1D), incorporates global context paths, encoding environmental dynamics into a modulation vector $g\in R^{H\times W\times C}$, where H=W=32 and C=64. This vector serves as a spatially adaptive gate applied channel-wise to the fused features, expressed as follows:

(1) $$F_{fused}\left(i,j,c\right)=g\left(i,j,c\right)\cdot \left(\alpha F_{RGB}\left(i,j,c\right)+\left(1-\alpha \right)F_{IR}\left(i,j,c\right)\right),$$

where (i,j) denotes the spatial position, *c* is the channel index, α∈[0,1] is a learnable initial modality weighting coefficient, and $F_{RGB}$ and $F_{IR}$ denote the feature maps of the RGB and infrared images, respectively. The modulation vector *g* enables fine-grained spatial adjustment of the fused output under different environmental conditions, suppressing noise responses due to low light or thermal interference. Theoretically, this mechanism can be interpreted as a combination of state space modeling and modality attention. The state space component offers global modeling capabilities for sequential environmental inputs, abstractly represented by

(2) $$h_{t}=Ah_{t-1}+Bx_{t},\;y_{t}=Ch_{t},$$

where *A*, *B*, and *C* are trainable matrices, $x_{t}$ is the environmental input, $h_{t}$ is the hidden state, and $y_{t}$ is the output guidance signal. With the integration of nonlinear activation, this structure provides strong fitting capabilities for complex sequences with periodicity or abrupt changes. When combined with the modality fusion layer, it enables flexible and precise modulation of attention weights. Within the overall framework, this mechanism operates in conjunction with the cross-modal attention feature fusion module. While the latter ensures spatial and semantic alignment across visual modalities, the former dynamically regulates modality-level contributions in response to environmental variations. This top-down and bottom-up synergy facilitates stable and distinctive target detection in complex multimodal co-recognition scenarios.

#### 3.3.4. Decoupled Dual-Target Detection Head

**Figure 5:**
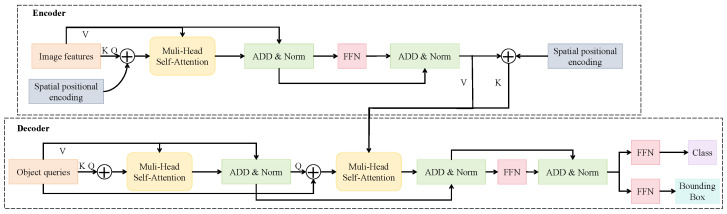
Architecture of the decoupled dual-target detection head. This module is designed to address the heterogeneity between pest and predator targets, employing separate attention decoding paths for modeling category and spatial information. Multi-head self-attention and positional encoding are used to generate class probabilities and bounding box parameters independently for each target type, enhancing small object separation and category-specific recognition.

To address challenges such as high visual similarity, significant scale variation, and class imbalance between pest and predator targets in field environments, a decoupled dual-target detection head module was designed to independently model pest and predator detection tasks and enhance task-specific feature representation. As shown in Figure 5, this module is inspired by the DETR architecture, incorporating a Transformer-based object query mechanism. Multimodal fused features are fed into the encoder, along with two distinct sets of decoupled queries, each responsible for detecting either pest or predator targets. Structurally, the module consists of an encoder, decoder, and two separate detection heads (Head-Pest and Head-Predator). The encoder is composed of N=6 stacked Transformer blocks, each containing a multi-head self-attention module (with $d_{model}=256$, h=8) and a feed-forward network consisting of two linear layers with dimensions 256→1024→256. The decoder similarly comprises M=6 layers, each including cross-attention between object queries and image features, multi-head self-attention, and a feed-forward network, ultimately producing independent object embeddings for the two query sets.

Each object query is represented as $Q\in R^{N_{q}\times d_{model}}$, where $N_{q}=100$ denotes the number of queries. In the detection head, separate feed-forward networks (FFNs) are constructed for pest and predator branches to perform bounding box regression and class probability prediction. Each branch consists of a three-layer MLP: one for bounding box regression with dimensions 256→256→4 and one for classification with dimensions 256→256→C, where C=8 denotes the number of target categories. Bounding box outputs are normalized to the range [0,1] using a sigmoid activation function, while class scores are obtained using a softmax function. The module is trained using a matching loss based on Hungarian assignment between object queries and ground-truth targets. The overall loss function is defined as follows:

(3) $$L=\lambda_{cls}\cdot L_{cls}+\lambda_{bbox}\cdot L_{bbox}+\lambda_{giou}\cdot L_{giou},$$

where $L_{cls}$ denotes the cross-entropy classification loss, $L_{bbox}$ is the $L_{1}$ loss for bounding box regression, and $L_{giou}$ represents the generalized IoU loss. The coefficients $\lambda_{cls}$, $\lambda_{bbox}$, and $\lambda_{giou}$ are weighting factors for balancing the loss components. To mitigate the issue of class imbalance between pest and predator categories, a class weighting factor $\omega_{i}$ is incorporated into $L_{cls}$ as follows:

(4) $$L_{cls}=-\sum_{i=1}^{C}\omega_{i}\cdot y_{i}\log\left(p_{i}\right),$$

where $y_{i}$ is the ground-truth label, $p_{i}$ is the predicted probability, and $\omega_{i}$ is the inverse frequency-based class weight. This decoupled detection structure is designed to operate in conjunction with the environment-guided modality attention mechanism. While the latter provides adaptive weighting of visual modalities during feature fusion based on environmental conditions, the former focuses on task-specific semantic differentiation and attribute modeling during prediction. Together, these components establish a closed-loop architecture from modality alignment to task decoupling. Compared to traditional unified detection heads, the proposed structure significantly improves the detection of small objects and enhances class-specific representation. This design effectively reduces misclassification caused by visual similarity between pests and predators and demonstrates superior accuracy and mAP performance, particularly in scenarios involving class imbalance or dense object coexistence, highlighting its strong practical applicability and generalization potential.

## 4. Results and Discussion

### 4.1. Experimental Setup

#### 4.1.1. Evaluation Metrics

To comprehensively evaluate the detection performance of the proposed multimodal recognition model for pests and their predators under complex field conditions, four widely used object detection metrics were adopted: precision, recall, F1-score, and mean average precision at an IoU threshold of 0.5 (mAP50). The mathematical definitions of these metrics are given as follows:

(5) $$\mathrm{Precision}=\frac{TP}{TP+FP}$$

(6) $$\mathrm{Recall}=\frac{TP}{TP+FN}$$

(7) $$F1-\mathrm{Score}=\frac{2\cdot \mathrm{Precision}\cdot \mathrm{Recall}}{\mathrm{Precision}+\mathrm{Recall}}$$

(8) $$\mathrm{IoU}=\frac{\mathrm{Area}(B_{p}\cap B_{gt})}{\mathrm{Area}(B_{p}\cup B_{gt})}$$

(9) $$\mathrm{mAP}@50=\frac{1}{N}\sum_{i=1}^{N}{\mathrm{AP}}_{i}^{\mathrm{IoU}=0.5},$$

where TP (true positive) denotes the number of correctly predicted target instances, FP (false positive) indicates the number of non-target samples incorrectly predicted as targets, and FN (false negative) refers to the number of ground-truth targets missed by the model. $B_{p}$ and $B_{gt}$ represent the predicted and ground-truth bounding boxes, respectively. $\mathrm{Area}(B_{p}\cap B_{gt})$ and $\mathrm{Area}(B_{p}\cup B_{gt})$ denote the intersection and union areas, respectively. Intersection over Union (IoU) serves as a critical metric for measuring the overlap between predicted and ground-truth boxes. ${\mathrm{AP}}_{i}^{\mathrm{IoU}=0.5}$ refers to the average precision of class *i* at an IoU threshold of 0.5, and *N* denotes the total number of categories (two in this study: pests and predators).

#### 4.1.2. Platform Configuration

All experiments were conducted on a unified hardware platform to ensure result consistency and stability. Specifically, model training and testing were performed on a workstation equipped with an Intel i7 processor and 8 GB of RAM, while inference performance was evaluated on the NVIDIA Jetson Xavier manufactured in China at factories operated by Foxconn (Hon Hai Precision Industry Co., Ltd.) edge computing platform. This platform provides strong embedded computational capability and is suitable for deploying multimodal recognition models in real-world agricultural monitoring applications. Such a configuration ensures a balanced evaluation of the model’s performance under both development and deployment conditions.

#### 4.1.3. Baseline Models

To validate the effectiveness of the proposed multimodal recognition framework in the joint detection of pests and their predators, eleven representative models were selected as baseline methods: YOLOv5 [36], YOLOv8 [37], YOLO-YSTs [17], RetinaNet [38], Faster R-CNN [39], SSD (Single Shot MultiBox Detector) [40], DETR [41], Sparse R-CNN [42], EfficientDet-D3 [43], early fusion, late fusion, and cross-modal attention fusion. These baselines encompass both single-stage and two-stage paradigms, as well as convolution-based and Transformer-based architectures, covering a wide range of accuracy–speed trade-offs and model complexities. YOLOv5, with its mature and stable architecture, is widely used for embedded and edge deployments. YOLOv8 provides strong real-time performance while maintaining competitive accuracy across diverse detection tasks. RetinaNet [38] employs focal loss to mitigate class imbalance, enhancing performance for small-object detection. YOLO-YSTs, based on YOLOv10n, is optimized for small and overlapping targets and achieves a favorable balance between detection accuracy and inference speed. Faster R-CNN [39], as a classical two-stage detector, offers precise localization and flexible feature modeling, supporting deeper analysis of modality contributions. SSD presents a compact and efficient architecture suitable for real-time applications on resource-constrained devices. DETR leverages a Transformer encoder–decoder to model global context, improving detection in cluttered backgrounds. Sparse R-CNN uses sparse queries to enhance the detection of small and medium objects. EfficientDet-D3 combines EfficientNet and BiFPN to achieve high accuracy with relatively low computational cost, making it promising for embedded scenarios. The early fusion baseline concatenates RGB, thermal, and environmental features at the input level for unified processing, while the late fusion baseline aggregates the outputs of independent single-modal detectors to preserve modality-specific learning. The cross-modal attention fusion baseline enables interaction between RGB and thermal features through attention layers before integration with environmental features, facilitating the use of complementary information.

### 4.2. Overall Performance Comparison of Detection Models on Multimodal Pest and Predator Recognition Tasks

This experiment was designed to systematically evaluate the performance differences among mainstream object detection models in the context of multimodal pest and predator recognition, thereby validating the effectiveness and robustness of the proposed method in complex agricultural scenarios. Unlike traditional unimodal detection tasks, the current experimental setting integrates RGB images, infrared thermal imaging, and environmental sensor data, posing a challenge to the models’ ability to integrate multi-source information and distinguish fine-grained targets. Evaluation metrics included precision, recall, F1-score, and mAP@50, providing a comprehensive assessment of accuracy, coverage, and overall stability.

**Figure 6:**
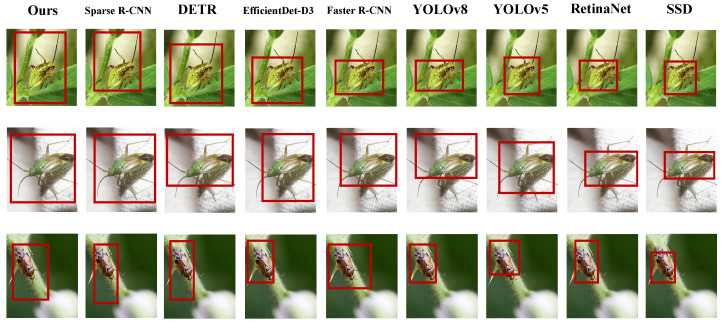
Visualization of detection results across different models on real-world field samples.

**Figure 7:**
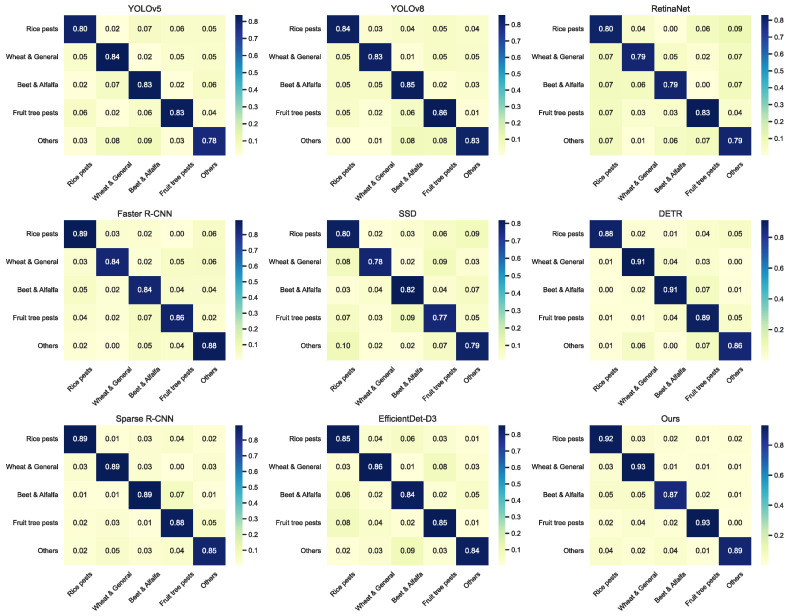
Comparative confusion matrices of mainstream object detection models across five pest and predator categories.

As shown in Table 3 and Figure 6 and Figure 7, YOLO-based models, due to their lightweight structure, exhibit fast inference but limited handling of small and overlapping targets. One-stage detectors such as RetinaNet and SSD demonstrate relatively lower accuracy when processing high-density small objects, indicating limited discriminative ability under complex backgrounds. In contrast, models like Faster R-CNN and EfficientDet-D3 benefit from feature pyramid and multi-scale fusion strategies, yielding more stable performance. Transformer-based architectures, such as DETR and Sparse R-CNN, exhibit superior context modeling capabilities, achieving higher mAP and F1 scores compared to conventional CNN-based models. From a mathematical perspective, models like YOLO and SSD fundamentally rely on anchor-based local convolution features and lack global modeling capacity, making it challenging to capture semantic relationships across modalities in high-dimensional joint feature spaces. Although RetinaNet mitigates class imbalance through focal loss, its feature contrastive ability remains limited, leading to misclassification between visually similar pests and predators. While Faster R-CNN improves candidate region filtering via region proposal mechanisms, its convolutional receptive field still struggles with modality-specific adaptation between infrared and RGB inputs. DETR and Sparse R-CNN, by leveraging Transformer-based positional attention, effectively capture both local and global dependencies, making them more suitable for handling non-aligned semantic cues across modalities. The proposed model incorporates cross-modal attention fusion, environment-guided mechanisms, and decoupled recognition heads, forming a closed-loop structure from modality alignment and dynamic reweighting to target-specific discrimination. This is mathematically reflected in multi-level transformations and attention-based compression across the input feature space, contributing to notable improvements in both accuracy and robustness. The superior performance of our model can be attributed to three main factors: the cross-modal attention mechanism effectively aligns RGB and thermal features, with environment-guided weighting adaptively adjusting the fusion ratio; the multi-scale feature enhancement module improves detection of small and multi-scale targets; and the contrastive loss strengthens feature separation between pests and predators, reducing misclassification. The synergy of these modules yields significant overall performance gains.

### 4.3. Ablation Study on Module-Level Contributions in the Multimodal Recognition Framework

This experiment was conducted to validate the actual contribution of each core module in the proposed multimodal recognition framework. By incrementally removing the cross-modal attention, environment-guided mechanism, modality reweighting strategy, and decoupled recognition head, variations in precision, recall, F1-score, and mAP@50 were observed to reveal the importance of each component in modality alignment, feature selection, and task representation.

As presented in Table 4, the full model consistently outperforms all ablated variants across all metrics, with mAP@50 reaching 88.0%, while all other versions fall below 73%. These results indicate that the removal of any single module leads to significant performance degradation, especially under cross-modal conditions, where the model’s ability to handle input inconsistency and target diversity is substantially impaired. Theoretically, the cross-modal attention module enables semantic fusion between non-aligned RGB and infrared regions; its removal results in the loss of fine-grained spatial sensitivity and overall semantic coherence. The environment-guided mechanism adjusts modality trust dynamically based on external factors such as temperature, humidity, and illumination; its absence disables adaptive modality adjustment, particularly under extreme conditions such as nighttime or reflective surfaces. The modality reweighting strategy enhances the representation of informative channels; without it, noise interference increases and suppresses effective features. The decoupled recognition head targets class-specific discrimination between pests and predators; its removal results in confusion under high similarity and imbalanced distribution. Mathematically, these modules operate, respectively, on modality space mapping, guidance weight calculation, attention function modeling, and classification loss restructuring, collectively forming a synergistic loop to ensure the model’s completeness and robustness in fusion, representation, and recognition.

### 4.4. Impact of Modality Ablation on Recognition Performance

This experiment was designed to evaluate the specific contribution of multimodal information to pest and predator recognition and to analyze the influence of different modality combinations on detection performance. By progressively removing thermal infrared images and environmental sensor data, the variations in core metrics such as precision, recall, F1-score, and mAP@50 were assessed to validate the practical value of multi-source data fusion in complex agricultural scenarios.

As presented in Table 5, the model achieved the highest performance when all three modalities—RGB, thermal infrared, and environmental sensors—were jointly used, with mAP@50 reaching 88.0%. Removing the sensor data while retaining RGB and thermal inputs led to a performance drop to 80.2%. When only RGB and sensor data were used, the mAP decreased further to 78.1%, and when limited to RGB images alone, performance was lowest, with mAP at only 75.4%. This trend demonstrates the complementary nature of the three modalities. Particularly under complex backgrounds and for small-object detection, both infrared and environmental data provided critical support. From the perspective of model design and mathematical formulation, RGB images contain rich visual cues but face limitations under conditions such as uneven illumination or occlusion. Thermal infrared imagery compensates for RGB weaknesses in low-light environments, while environmental sensor data introduce exogenous variables to guide attention reweighting mechanisms. The model maps all modalities into a unified high-dimensional feature space through dedicated encoders and employs attention-based fusion to assign dynamic weights across modalities, enabling cross-modal enhancement. The removal of infrared or sensor modalities weakens this context-aware modeling ability, thereby degrading feature fusion effectiveness and resulting in substantial accuracy loss. This confirms the irreplaceable mathematical value of multimodal features in suppressing redundancy, adapting to environmental variations, and aligning target semantics, establishing them as a core factor in enhancing recognition performance in complex field scenarios.

### 4.5. Inference Efficiency and Model Size Comparison on Edge Platforms

This experiment evaluates the inference efficiency, deployment feasibility, and accuracy of all baseline models and the proposed method on two representative edge computing platforms: Jetson Xavier and Raspberry Pi 4B. The evaluation considers three metrics: model size, frames per second (FPS), and detection accuracy (mAP@50). These results reflect both resource consumption under deployment constraints and the trade-offs between compactness and detection performance.

As shown in Table 6 and Table 7, YOLOv5 and YOLOv8 achieved the highest inference speeds of 34.6 FPS and 36.8 FPS, respectively, making them suitable for real-time applications. However, their mAP scores of 76.9% and 79.5% reveal an inherent limitation in detection precision. Faster R-CNN and DETR yielded higher accuracies of 81.2% and 82.9%, respectively, but their large model sizes (over 165 MB) resulted in slower inference speeds of only 12.1 FPS and 10.4 FPS, making real-time deployment on embedded devices impractical. EfficientDet-D3 demonstrated a more balanced trade-off between model size and accuracy. The proposed model achieved a superior mAP of 88.0%, with a compact model size of 48.3 MB and a practical inference speed of 25.7 FPS, outperforming all other models in overall efficiency. From a structural and mathematical perspective, YOLO-based models adopt a single-stage detection architecture characterized by compactness and speed, relying on anchor regression and local convolutional features. However, their limited capacity for global modeling constrains their ability to fuse semantic cues across modalities, thereby capping their accuracy. Faster R-CNN and DETR introduce region proposals and global attention mechanisms, which allow for better contextual and long-range dependency modeling, thus yielding higher precision in multimodal scenarios. However, their architectures inherently involve extensive fully connected computations and cross-layer feature interactions, resulting in significant model size and computational overhead. In contrast, the proposed method incorporates cross-modal feature compression and modality-guided mechanisms to reduce redundant multimodal expansions. A lightweight Transformer module replaces traditional global attention, retaining cross-modal modeling capacity while controlling computational costs. This design enables a high-performance balance across accuracy, size, and speed, making it particularly suited for resource-constrained agricultural edge deployment.

### 4.6. Performance Evaluation Under Different Time-of-Day and Weather Conditions

To evaluate the effectiveness of the environment-guided attention mechanism under varying environmental conditions, the test set was partitioned by time-of-day (morning/noon/evening) and weather conditions (sunny/cloudy/foggy), and each subset was evaluated independently. The results in Table 8 show that our method consistently outperforms the baseline without environment guidance (w/o EGA) across all scenarios. Notably, in the “evening” condition with rapid lighting changes and in the “foggy” condition where thermal imaging dominates, mAP@50 improved by 3.1% and 4.5%, respectively. This confirms that the environment-guided attention adaptively adjusts multimodal fusion weights based on external conditions, improving detection performance and stability in complex environments.

### 4.7. Discussion

#### 4.7.1. Environmental Robustness and Deployability

The proposed multimodal pest and predator recognition method demonstrated notable performance and deployability in complex field environments. Its core advantage lies in the integration of RGB images, thermal infrared imaging, and environmental sensor data, including temperature, humidity, and light intensity, enabling the extraction of more discriminative target features from multi-source inputs and enhancing adaptability to dynamic agricultural conditions. In real-world agricultural scenarios, pest monitoring often encounters significant challenges, such as light spot interference under intense sunlight during the day, image degradation under low-light conditions at night, and lens fogging under high-temperature and high-humidity conditions. Traditional single-modality recognition models are prone to false positives or missed detections under such circumstances, severely affecting the accuracy of downstream pest control strategies. The proposed method addresses these limitations by introducing thermal infrared images, which provide stable thermal response signals and allow for the clear delineation of target boundaries even under extreme lighting conditions. Simultaneously, environmental sensor data act as auxiliary variables to guide the model in dynamically adjusting its attention across different modalities, thereby significantly improving environmental robustness and recognition stability. In practical applications, the method has been proven deployable on edge devices such as Jetson Xavier and Raspberry Pi equipped with AI acceleration modules. It can be integrated into small-scale field inspection robots, intelligent pest trapping devices, or autonomous spraying systems, enabling low-cost, high-frequency co-recognition of pests and predators. This is particularly critical for the advancement of green agriculture. In organic farming systems where pesticide usage is restricted, the accurate identification of predator species and their distribution densities provides essential guidance for precision release and biological control decisions. For example, in greenhouse vegetable production in Shouguang, Shandong Province, where diurnal temperature variation and lighting conditions fluctuate significantly during spring and autumn, the proposed model can stably detect noctuid moths and green lacewings, facilitating zone-specific predator release via automated dispensing systems. Similarly, in maize and legume cultivation regions such as Bayannur, Inner Mongolia, the multimodal recognition terminals integrated with the proposed model enable routine field monitoring and maintain high detection accuracy even under low-light conditions at dusk, reliably identifying pests such as armyworms and lady beetles. These capabilities provide robust data support for regional pest forecasting and agricultural scheduling.

#### 4.7.2. Practical Deployment and Application Considerations

For practical implementation in field pest management, the developed multimodal recognition model can be seamlessly integrated into intelligent agricultural devices equipped with RGB and thermal infrared cameras as well as environmental sensors. During operation, multi-source data are synchronously collected and preprocessed on site, including illumination normalization for RGB frames, noise suppression for thermal imagery, and real-time calibration of temperature, humidity, and light intensity readings. These inputs are fed into the independent encoders for each modality, followed by the environment-guided attention fusion module to dynamically balance modality contributions under varying field conditions. The fused features are processed by the detection head to generate pest and predator bounding boxes and classifications, which are subsequently transmitted to a local or remote control terminal. Based on the recognition results, automated devices—such as precision sprayers, smart traps, or predator release systems—can execute targeted interventions while logging detection records for historical analysis and pest forecasting. On edge computing platforms such as Jetson Xavier and Raspberry Pi, the complete pipeline can run in real time with low power consumption, enabling continuous, unattended monitoring in large-scale agricultural deployments.

### 4.8. Limitation and Future Work

Despite the superior recognition performance and environmental adaptability demonstrated by the proposed multimodal pest and predator recognition framework, several limitations remain. First, the current model primarily relies on fixed-position multisensor platforms, which are constrained by acquisition angles and field-of-view limitations. This poses a risk of missed detections for concealed targets in tall crops or large-scale fields. Future research should explore the integration of mobile acquisition platforms, such as unmanned aerial vehicles (UAVs) and multi-joint robotic arms, to expand the spatial perception capability of the model. Second, in terms of data coverage, the current multimodal dataset was collected mainly from representative regions (Shandong and Inner Mongolia), lacking samples from tropical and subtropical climates. As a result, the regional generalization capacity of the model requires further validation and enhancement. Future efforts will focus on incorporating techniques such as model distillation, quantization, and hierarchical inference to improve computational efficiency and response speed under resource-constrained conditions.

## 5. Conclusions

Against the backdrop of rapidly advancing smart agriculture, the development of pest and predator recognition models with strong environmental adaptability and high accuracy has become crucial for enhancing green control practices and promoting sustainable agroecological development. To address the limitations of existing approaches that exhibit strong reliance on single modalities and unstable recognition performance under complex field conditions, a multimodal recognition framework was proposed. This framework integrates RGB images, thermal infrared maps, and environmental sensor data and incorporates a cross-modal attention mechanism, an environment-guided modality weighting strategy, and a decoupled recognition head design. These components collectively enhance the model’s capability to distinguish complex targets and improve cross-modal collaborative perception. Extensive experimental evaluations demonstrated that the proposed method significantly outperforms several mainstream detection models across key performance metrics, including precision, recall, F1-score, and mAP@50. Under full multimodal integration, the model achieved a precision of 91.5%, a recall of 89.2%, an F1-score of 90.3%, and a mAP@50 of 88.0%, all markedly superior to baseline models such as YOLOv8, DETR, and EfficientDet-D3. Furthermore, module-level and modality-level ablation studies validated the critical role of the cross-modal attention mechanism and environment-guided strategies in enhancing recognition robustness and fine-grained target discrimination. The model was also evaluated on the Jetson Xavier edge platform, demonstrating favorable computational efficiency and deployment feasibility in real-world agricultural settings.

## Figures and Tables

**Table 1:** Summary of the collected multimodal dataset.

Modality	Quantity	Resolution/Format	Description
RGB images	3249	1920 × 1080	Captured under varying lighting conditions
Infrared images	1210	640 × 480	Synchronized with RGB frames
Temperature and humidity	720,000	Temp + RH	1 Hz, spanning two sites over five months
Light intensity	720,000	Single-channel LDR	1 Hz, aligned with image timestamps

**Table 2:** Pest and predator species counts, host crops, time of day, and weather coverage in the dataset.

Facet	Category	Count (Images)
Species classification	Pest species	6
	Predator species	2
Host crops	Rice	1200
	Wheat	1050
	Citrus	999
Time of day	Morning	1050
	Noon	1099
	Evening	1100
Weather	Sunny	1300
	Cloudy	1049
	Foggy	900

**Table 3:** Overall performance comparison of different detection models in multimodal pest and predator recognition.

Model	Precision (%)	Recall (%)	F1-Score (%)	mAP@50 (%)
YOLOv5	82.4	78.6	80.4	76.9
YOLOv8	84.2	81.0	82.6	79.5
YOLO-YSTs	86.5	83.4	84.9	81.8
RetinaNet	80.1	76.7	78.3	74.8
Faster R-CNN	85.7	82.3	84.0	81.2
SSD	78.3	74.5	76.3	71.6
DETR	87.1	84.2	85.6	82.9
Sparse R-CNN	88.4	85.1	86.7	84.1
EfficientDet-D3	86.0	82.8	84.3	81.7
Early Fusion	88.7	86.0	87.3	84.8
Late Fusion	89.3	86.5	87.9	85.5
Cross-Modal Attention	90.2	87.5	88.8	86.7
**Ours**	**91.5**	**89.2**	**90.3**	**88.0**

**Table 4:** Module-level ablation study for the proposed multimodal recognition model.

Setting	Precision (%)	Recall (%)	F1-Score (%)	mAP@50 (%)
A. Full Model (All Modules)	**91.5**	**89.2**	**90.3**	**88.0**
B. w/o Cross-Modal Attention	78.4	74.6	76.4	72.1
C. w/o Environment-Guided Attention	79.2	75.3	77.2	73.5
D. w/o Modality Reweighting	77.8	73.1	75.3	71.8
E. w/o Decoupled Detection Head	78.6	74.2	76.3	72.7

**Table 5:** Impact of modality ablation on recognition performance.

Modality Configuration	Precision (%)	Recall (%)	F1-Score (%)	mAP@50 (%)
RGB + Thermal + Sensor (Full)	**91.5**	**89.2**	**90.3**	**88.0**
RGB + Thermal only	84.7	81.5	83.1	80.2
RGB + Sensor only	82.9	78.6	80.7	78.1
RGB only	80.3	75.8	77.9	75.4

**Table 6:** Inference efficiency and model size comparison on Jetson Xavier.

Model	FPS	Model Size (MB)	mAP@50 (%)
YOLOv5	34.6	22.4	76.9
YOLOv8	36.8	25.7	79.5
YOLO-YSTs	33.2	28.4	81.8
RetinaNet	27.5	145.2	74.8
Faster R-CNN	12.1	174.6	81.2
SSD	29.7	98.4	71.6
DETR	10.4	165.9	82.9
Sparse R-CNN	11.6	172.3	84.1
EfficientDet-D3	28.3	52.1	81.7
Early Fusion	27.8	50.2	84.8
Late Fusion	26.9	49.8	85.5
Cross-Modal Attention	26.2	48.9	86.7
**Ours**	**25.7**	**48.3**	**88.0**

**Table 7:** Inference efficiency and model size comparison on Raspberry Pi 4B.

Model	FPS	Model Size (MB)	mAP@50 (%)
YOLOv8	13.8	25.7	79.5
YOLOv5	12.9	22.4	76.9
YOLO-YSTs	12.1	28.4	81.8
RetinaNet	9.6	145.2	74.8
Faster R-CNN	4.2	174.6	81.2
SSD	10.8	98.4	71.6
DETR	3.9	165.9	82.9
Sparse R-CNN	4.1	172.3	84.1
EfficientDet-D3	10.4	52.1	81.7
Early Fusion	10.1	50.2	84.8
Late Fusion	9.8	49.8	85.5
Cross-Modal Attention	9.6	48.9	86.7
**Ours**	**9.4**	**48.3**	**88.0**

**Table 8:** Performance comparison under different time-of-day and weather conditions.

Condition	Method	mAP@50 (%)	F1-Score (%)
Morning	w/o EGA	86.2	84.7
	Proposed (EGA)	**88.5**	**86.4**
Noon	w/o EGA	87.5	85.9
	Proposed (EGA)	**89.1**	**87.0**
Evening	w/o EGA	84.3	82.5
	Proposed (EGA)	**87.4**	**85.2**
Sunny	w/o EGA	88.0	86.2
	Proposed (EGA)	**90.1**	**87.8**
Cloudy	w/o EGA	85.7	83.9
	Proposed (EGA)	**88.0**	**85.6**
Foggy	w/o EGA	82.1	80.0
	Proposed (EGA)	**86.6**	**83.4**

## Data Availability

The original contributions presented in this study are included in the article. Further inquiries can be directed to the corresponding author.
